# Initial development of skill with a reversed bicycle and a case series of experienced riders

**DOI:** 10.1038/s41598-024-54595-8

**Published:** 2024-02-21

**Authors:** Justine Magnard, Timothy R. Macaulay, E. Todd Schroeder, Christopher Laine, James Gordon, Nicolas Schweighofer

**Affiliations:** 1 Biokinesiology and Physical Therapy, University of Southern California, Los Angeles, CA USA; 2 Faculté des Sciences de la Motricité, Université Libre de Bruxelles, Bruxelles, Belgium; 3 KBR, Houston, TX USA; 4 Occupational Science and Occupational Therapy, University of Southern California, Los Angeles, CA USA

**Keywords:** Learning and memory, Motor control, Human behaviour

## Abstract

Riding a bicycle is considered a durable skill that cannot be forgotten. Here, novice participants practiced riding a reversed bicycle, in which a reversing gear inverted the handlebar’s rotation. Although learning to ride the reversed bicycle was possible, it was slow, highly variable, implicit, and followed an S-shape pattern. In the initial learning phase, failed attempts to ride the normal bicycle indicated strong interference between the two bicycle skills. While additional practice decreased this interference effect, a subset of learners could not ride either bicycle after eight sessions of practice. Experienced riders who performed extensive practice could switch bicycles without failed attempts and exhibited similar performance (i.e., similar handlebar oscillations) on both bicycles. However, their performance on the normal bicycle was worse than that of the novice bicycle riders at baseline. In conclusion, “unlearning” of the normal bicycle skill precedes the initial learning of the reversed bicycle skill, and a signature of such unlearning is still present following extensive practice.

## Introduction

Riding a bicycle is the quintessential motor skill. It is challenging to acquire, but once learned, the skill is highly durable and apparently resistant to forgetting. The phrase “just like riding a bike” refers to any skill which, once learned, is not forgotten. Learning to ride a bike involves acquiring tacit knowledge^1^ stored in the procedural memory system. Skilled riders can balance and steer the bicycle without explicit knowledge or awareness of the complex physical rules they must obey to balance and steer the bike^2,3^.

The fundamental task to be learned in bicycle riding is maintaining balance as one pedals forward. As the bicycle tilts to one side or the other, the novice rider must learn to make a small turn of the front wheel in the same direction as the bike is leaning. The resulting curved path of the bicycle generates a centrifugal force that tends to right the bicycle^4^. However, what is learned is not a simple rule^5,6^. The centrifugal restoring force is proportional to the square of the velocity and the instantaneous radius of the turn. Thus, to maintain balance with minimal oscillations in tilt and handlebar rotation, the rider must learn a non-linear, speed-dependent mapping of the handlebar rotations required to counteract the tilt^7,8^. Indeed, a characteristic feature of initial learning in bicycle riding is a rapid oscillatory back-and-forth turning of the handlebars^9,10^, as the learner initially overcorrects. A skilled rider has refined the complex sensorimotor mapping that rapidly processes a continuously changing array of sensory data from multiple channels (notably visual and vestibular) and transforms those data into a precisely modulated sequence of muscle contractions that produce smooth steering adjustments.

Billions of bicycle riders worldwide have managed to acquire this skill. But how flexible is that skill if the typical relationships between speed, leaning, and handlebar rotation are modified? Not as flexible as one might assume. People can certainly ride bicycles of different sizes or styles. However, if the handlebar is geared such that a clockwise turn rotates the wheel counterclockwise, the resulting “reversed” bicycle is initially unrideable, as a widely viewed YouTube video has shown^11^. For the rider, the modification seems simple; a lean of the bicycle to one side will now require a turn of the handlebars in the opposite direction– but the required movement pattern is fundamentally incompatible with their normal, automatic bicycle riding skill, resulting in substantial interference. This video and previous studies^12,13^ have shown that learning to ride a reversed bicycle is eventually possible. However, there have been no reports of a detailed characterization of the learning process associated with this paradigm.

Whereas motor learning is often studied with simple laboratory tasks in which one learns to compensate for artificial physical or visual perturbations, here, in contrast, we studied the acquisition of a complex skill (see also^14–24^), riding the reversed bicycle, a task that is incompatible with a previous learned skill, riding the normal bicycle. With this study, we aim at answering the following three major questions in motor learning research: first, what is the time course of such complex “reversed” skill learning? Specifically, does it follow a commonly observed negatively accelerated shape, such as in motor adaptation, or does it follow a positively accelerated shape, which has been proposed as the hallmark of skill learning, but only seldom been observed^25,26^. Second, do novice learners develop interference from the new skill to the old skill during learning? In particular, do these interferences lead to people to “unlearn” how to ride the normal bicycle? Third, can extensive practice lead to the expression of the two incompatible skills with little interference between the two?

We address these questions in two ways. First, to study the early phase of learning we conducted Experiment 1, in which we instructed 20 novice participants to practice riding the reversed-steering bicycle in eight practice sessions, while monitoring their daily performance and improvement. Second, to study the long-term phase of learning, we took advantage of the fact that three individuals (three authors of this paper: JM, ETS, TM) were extensively practicing the reversed bicycle as a hobby, allowing us to perform a natural experiment with a small convenient sample of four experienced riders. We measured the performance of experienced riders on both bicycles, their ability to switch from the reversed to the normal bicycle, and we then compared the performance of the novice and experienced riders on both bicycles. Performance was measured with both 20-m straight line test and via the angular handlebar rotation velocity, as recorded by a gyroscope placed on the handlebar.

## Results

### Subjects can learn to ride the reversed bicycle with extensive practice

**Figure 1:**
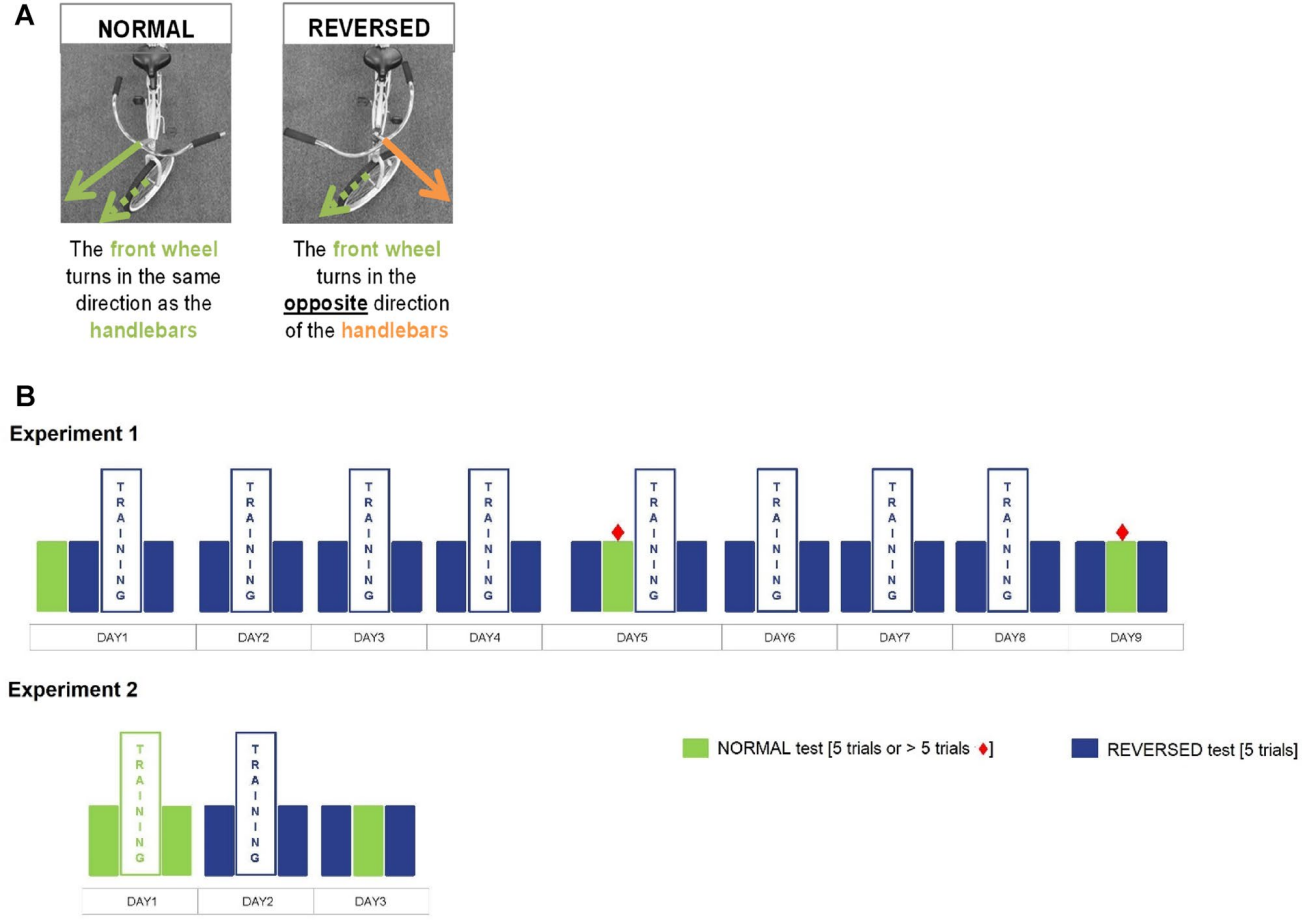
Experimental methods for Experiments 1 and 2. (**A**) Illustration of the normal and the reversed bicycle, characterized by a reversed handlebar-to-wheel coupling (*i.e*., when the handlebar is rotated right, the wheel turns left, and vice versa). (**B**) Top: schedule of practice and testing for the reversed and normal bicycles for the novice participants in Experiment 1. Before and after each practice session (DAY 1–DAY 8) and following the last practice session (DAY 9), the performance in riding the reversed bicycle was assessed in 5-trial tests (blue) in which we measured the distance ridden along a 20-m straight path. During the interference assessment on DAY 5 and DAY 9 (green), the instruction was to ride the normal bicycle for as many trials as needed to ride 20 m. ♦: indicates that more than 5 trials were allowed in these tests. All other tests comprised 5 trials. Bottom: schedule of training and testing for the reversed and normal bicycles for the Experienced participants in Experiment 2. Because there were no failed attempts, all tests comprised 5 trials.

Twenty novice participants in Experiment 1 practiced riding the reversed bicycle for 8 consecutive weekday practice sessions of 10 min each (Fig. 1B). Before and after each session (DAY 1–DAY 8) and following the last practice session (DAY 9), the participants completed pre- and post-practice tests of 5 trials in which we measured performance as the distance ridden along a 20-m straight path. A test trial started when the participant placed both feet on the pedals and ended when one foot touched the ground, with the objective to ride as far as possible, up to 20 m. In trials where participants traveled 20 m, performance was further assessed via the angular handlebar rotation velocity, as recorded by a gyroscope placed on the handlebar (see “Methods”). On DAY 1, 5, and 9, the participants were also tested on a normal bicycle, identical to the reversed bicycle except for the reversing gear.

**Figure 2:**
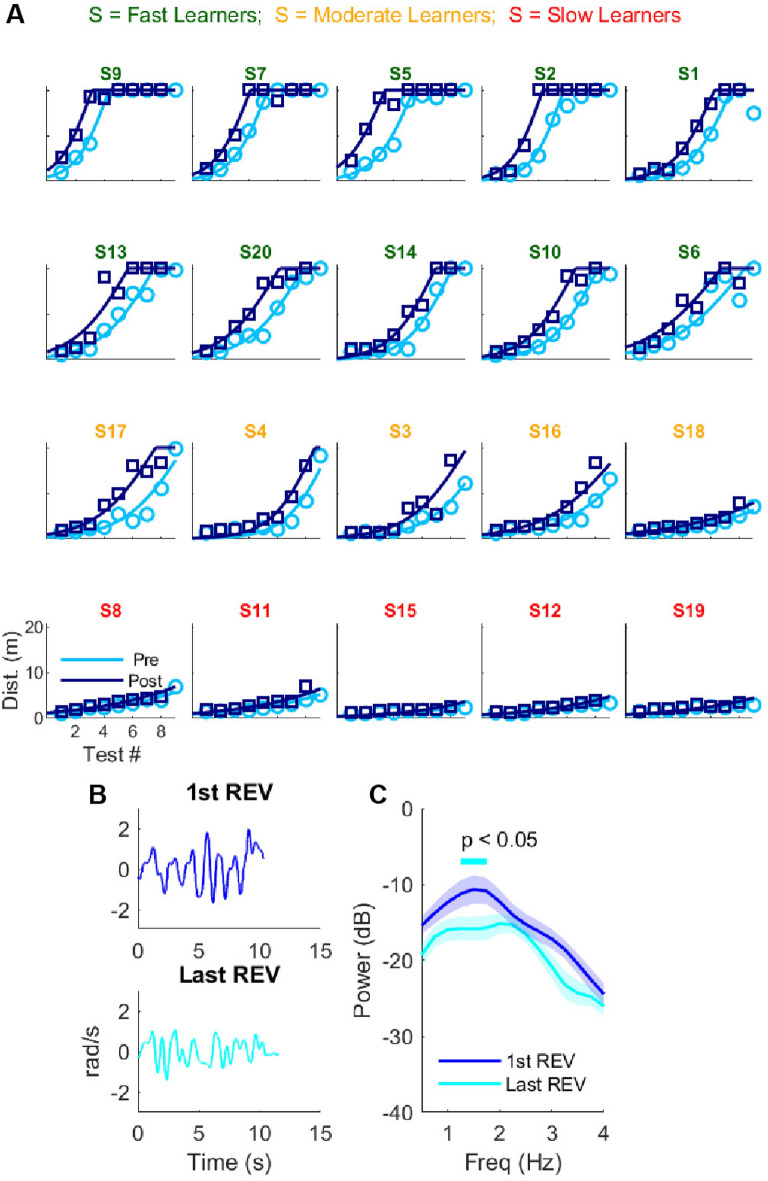
Learning the reversed bicycle in Experiment 1. (**A**) Average distance on the reversed bicycle in pre-tests (light blue circle) and post-tests (dark blue squares) and corresponding half-sigmoidal model fits for the 20 Novice participants of Experiment 1. The first 10 participants are classified as “Fast learners”, the next five as “Moderate learners”, and the last 5 as “Slow Learners”—see text for details. x-axis: test #. y-axis: distance in meters in the 20 straight line test. (**B**) Example of handlebar oscillations for the first successful trial on the reversed bicycle and the last successful trial on Day 9 for a representative Fast Learner participant. (**C**) Changes in power of handlebar oscillations between the first and last successful trials for the 10 Fast Learners. *REV* reversed bicycle.

With practice, the distance ridden improved over the 8 sessions overall (paired t-test: *t* < − 3.34, *p* < 0.05; mean distance DAY 1 *pre*-practice: 1.5 ± 0.3 m *vs*. mean distance DAY 9 *pre*: 14.2 ± 6.9 m; all results in mean ± SD; see Supplementary Table 1). However, despite being instructed and shown how to ride the reversed bicycle (i.e., to rotate the handlebar in the opposite direction to the tilt), no participant succeeded in riding the reversed bicycle by the end of DAY 1 (see Fig. 2A). Thus, knowledge of the reversed bicycle rule did not result in participants successfully riding the reversed bicycle on DAY 1, suggesting that learning how to ride the bicycle is an implicit learning process. Accordingly, we found no correlations between measures of cognitive interference, mental flexibility, and working memory with any metrics of reversed bicycle performance over time (see Supplementary material).

### The between-day change in performance typically followed an S-shaped pattern

The initial rate of change of performance appeared slow; then, for some riders it increased quickly in the following sessions (see Fig. 2A), suggesting an initial “discovery” phase. Indeed, test performance from all participants was well fitted with a non-linear mixed-effect model comprised of a lower-half sigmoidal model followed by a constant segment at 20 m (see Eq. (1) in “Methods” and Fig. 2A). This S-shaped, positively accelerated, model captured the initial slow phase followed by a rapid increase in performance for all participants and tests (see Fig. 2A; RMSE = 1.30 m). This contrasts with the poorer fit of a concave, negatively accelerated, exponential model, as often used in learning studies, e.g.,^27^, which does not include a discovery phase (RMSE = 2.25 m). We verified that the mean absolute value of the residuals of the exponential fits was larger than those of the sigmoidal model fit for all participants (a fair comparison because the models have the same number of parameters).

### Learning was highly variable between participants, with three distinct groups of learners

There was high variability on the day of the first improvement in performance, suggesting that a within-day discovery might be required before substantial across-day improvements were possible. If so, at the end of the discovery phase, the timing of the first within-day improvement (pre- vs. post-practice) should predict the timing of the first across-day improvement (change in pre-practice performance between consecutive days). We defined a threshold for ‘improvement’ as the first statistical deviation (z-score > 1.65) from the within-day improvements observed across all participants on DAY 1 and from the across-day improvements observed across all participants between DAY 1 and DAY 2. Supplementary Figure S1 shows that, indeed, the timing of the first within-day improvement is a strong predictor of the timing of across-day improvement (r = 0.92, p < 0.001).

Based on this threshold analysis, we identified three distinct sub-groups of participants. Fifteen of the 20 participants reached the phase of rapid improvement. Among these, 10 successfully rode 20 m in at least one pre-test trial by DAY 8 (mean 6.80 ± 0.36 days, see Supplementary Table 1). These are referred to as the Fast Learner group. The other 5 participants, referred to as the Moderate Learner group, were still improving on Day 9. The day on which performance reached the threshold was highly variable across the 15 participants in these two groups, ranging from 1 to 8 days (mean 3.8 days, SD 1.90). Five participants whose performance never reached the rapid improvement phase are referred to as the Slow Learner group. These data appear to indicate that these five participants were “non-learners”; however, interference results presented below will show that these participants were in the process of learning how to ride the reversed bicycle. Hence, we decided to call these participants “slow learners”.

Analysis of handlebar oscillations demonstrates that once the Fast Learners achieved the 20-m criterion, additional practice continued to improve performance via better control of the handlebar (Fig. 2B,C). Specifically, a comparison of the power spectra of the handlebar oscillations from 0.5 to 4 Hz between the first 20-m successful trial and the last trial on DAY 9 (see “Methods” and all individual data in Supplementary Fig. 2) shows that improvements due to additional practice led to significant reductions of power in 1.25–1.75 Hz handlebar oscillations (*p* < 0.05; paired-sample *t*-test with corrections for multiple comparisons via permutations; see “Methods”).

### Experienced riders can ride the reversed bicycle as skillfully as the normal bicycle

In Experiment 2, we tested whether extensive practice on the reversed bicycle allowed a separate group of four participants to ride the reversed bicycle as skillfully as the normal bicycle and whether they could switch between the normal and the reversed bicycle. The four participants practiced the reversed bicycle for a minimum of 12 h over 8 weeks. We then compared their skill in riding the reversed bicycle with their skill in riding the normal bicycle, using the angular handlebar rotation velocity, over three testing sessions on consecutive days (Fig. 1B). On DAY 1 and DAY 2, participants trained on the normal and reversed bicycle, respectively, for 10 min each and completed pre- and post-practice tests of 5 trials each. On DAY 3, we assessed the participants’ capacity to switch between bicycles, according to the design on DAY 9 in Experiment 1: 5 trials of the reversed bicycle, then 5 trials of the normal bicycle, and finally 5 trials on the reversed bicycle (see “Methods” for details).

**Figure 3:**
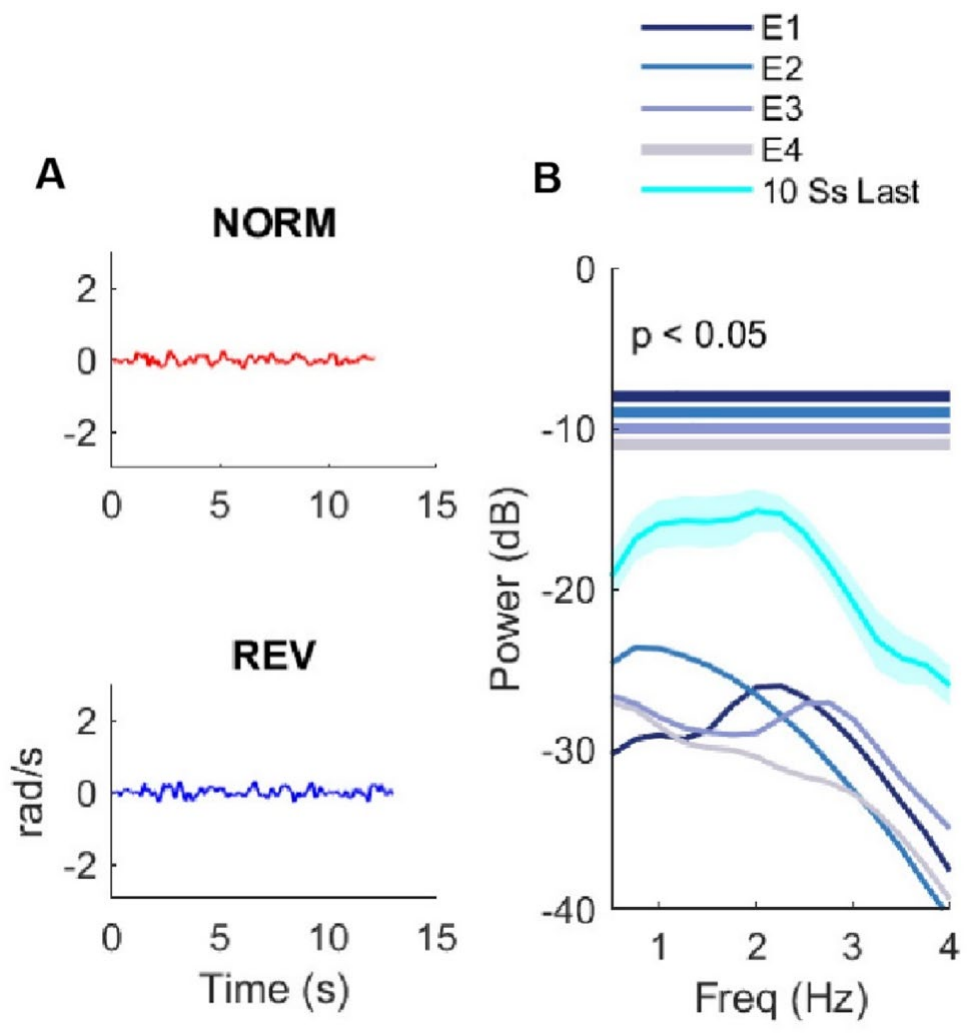
Skilled performance of experienced riders riding the reversed bicycle in Experiment 2. (**A**) Examples of handlebar oscillations for an Experienced rider riding both reversed and normal bicycles (reversed: last trial of Day 1; normal: last trial of Day 2). (**B**) Power spectra of handlebar oscillations. All four Experienced riders (E1-4) show lower power in all frequencies than the Fast Learners (10Ss). *REV* reversed bicycle, *NORM* normal bicycle.

The four participants of Experiment 2 can be characterized as “experienced” in riding the reversed bicycle for three reasons. First, on all testing days, they successfully rode both the reversed and the normal bicycles to the 20-m criterion in all trials without any failed attempts. Second, there was no difference between the reversed and the normal bicycle in the handlebar oscillations, as shown by the power of the oscillations at any frequencies between 0.5 and 4 Hz (*p* > 0.05, paired *t*-test with corrections for multiple comparisons via permutations, see “Methods”; see Fig. 3A for example of oscillations for one representative Experienced rider and Supplementary Fig. 3 for power of the handlebar oscillations for the four Experienced riders). Third, these participants were more skilled riders than the Fast Learners of Experiment 1 at the end of reversed bicycle practice (DAY 9), as shown by the smaller power of the handlebar oscillations at all frequencies (all p < 0.05; Fig. 3B).

### Learning to ride the reversed bicycle requires “unlearning” of normal bicycle riding in both Novice learners and Experienced riders

**Figure 4:**
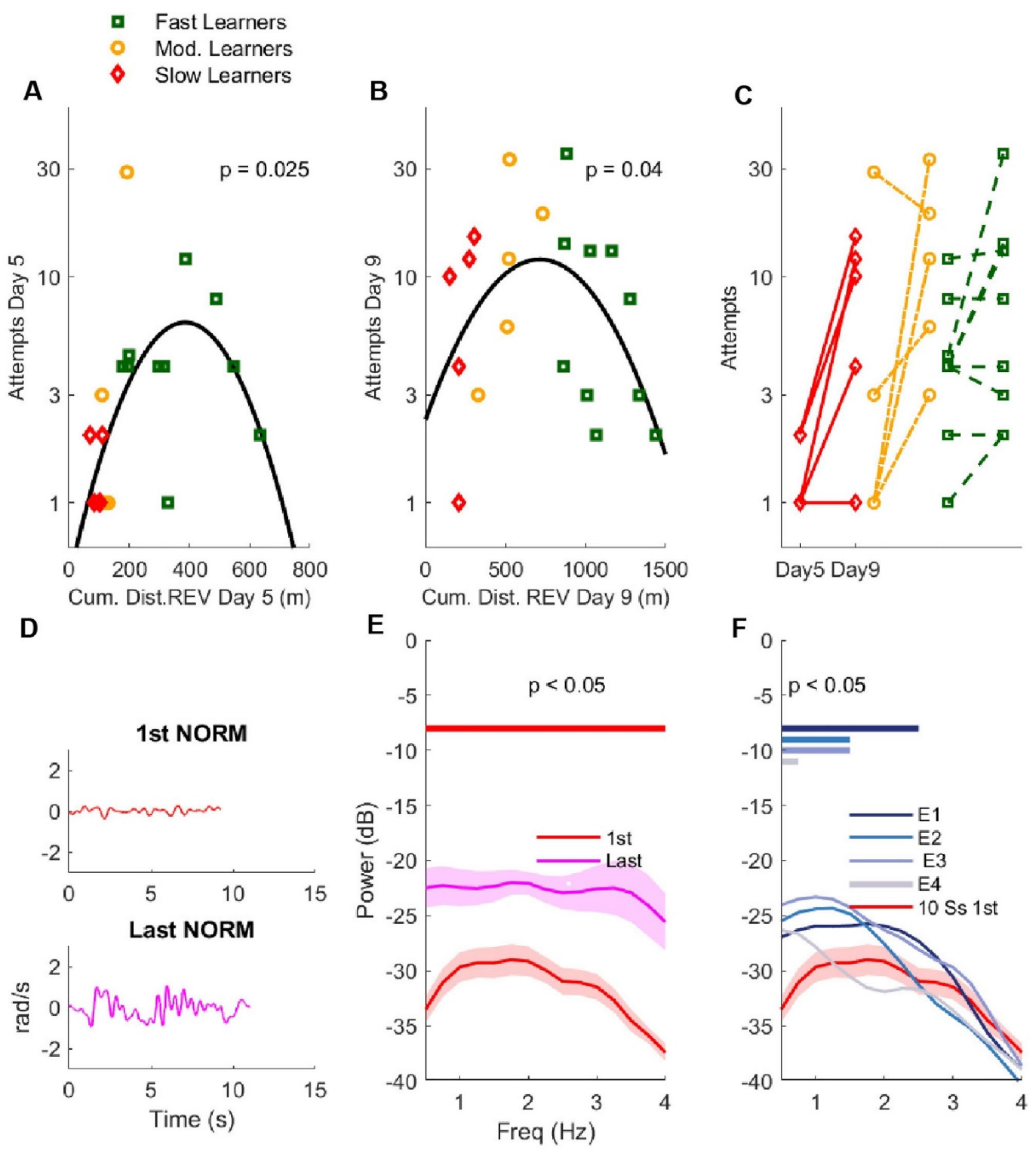
Unlearning how to ride the normal bicycle. Evidence from Experiments 1 and 2. The number of attempts to ride the normal bicycle shows an inverted U shape as a function of the cumulative distance in tests up to Day 5 (**A**) and Day 9 (**B**) (Experiment 1). (**C**) Number of attempts to ride the normal bicycle in Day 5 and Day 9 for all three groups. Note how, despite not being able to ride the reversed bicycle (Fig. 2A, last row), four out of five Slow Learners could not ride the normal bicycle at the end of practice with the reversed bicycle, as shown by the increase in the number of attempts from Day 5 to Day 9 (Experiment 1). (**D**,**E**) The Fast learners show larger oscillations amplitude (**D**) and power (**E**) on the normal bicycle at the end of practice than before practice (Experiment 1). (**F**) The four Experienced riders (E1-4) also show greater power in oscillations at low frequencies on the normal bicycle (Experiment 2) than the 10 Novices (Ss) on the normal bicycle on DAY 1 (Experiment 1).

We hypothesized that when the Novice participants of Experiment 1 were learning the reversed bicycle, they would have difficulties riding the normal bicycle due to the two tasks being physically mutually exclusive. In addition, because the degree of interference in motor tasks has been shown to decrease with extensive practice^28,29^, we predicted an inverted U relationship between the amount of practice on the reversed bicycle and the number of failed attempts before being able to ride the normal bicycle. We recorded the number of failed attempts on the normal bicycle on DAY 5 and DAY 9. On these days, following the 5 trials pre-test with the reversed bicycle, the Novice participants performed a second pre-test in which they attempted to ride the normal bicycle until they could ride 20 m (see Fig. 1A). Note that because we did not have a measure of the total distance ridden on the reversed bicycle during practice until DAY 5 and 9, we computed the cumulative distance on pre- and post-tests until these days as a proxy for this total distance. As predicted, a second-order (parabolic) model better fitted the (log_10_) number of failed attempts as a function of the cumulative distance on DAY 5 and DAY 9 than a first-order linear model (mixed effect models with subject random intercepts, *p* = 0.015, likelihood ratio test), showing an apparent inverted U curve for both days (Fig. 4A,B, significance of second order term at DAY 5, *p* = 0.025; at DAY 9, *p* = 0.40).

Whereas the Fast Learners required the same number of trials to ride the normal bicycle on DAY 5 and DAY 9 (*p* = 0.93, paired *t*-test), the Moderate and Slow Learners showed an increase in the number of failed attempts to ride the normal bicycle from DAY 5 to DAY 9 (paired *t*-test on log10 of attempts: p = 0.043). Strikingly, four of the five Slow Learners, despite not having reached the rapid phase of improvement with the reversed bicycle (see above), required more trials to ride 20 m with the normal bicycle on DAY 9 than on DAY 5 (an increase of 3, 8, 11, and 13 trials) (Fig. 4C). Thus, even though these participants showed little to no evidence that they were learning to ride the reversed bicycle, their ability to ride the normal bicycle was degraded. These results indicate that unlearning how to ride the normal bicycle *precedes* improvement in performance in how to ride the reversed bicycle.

Although the Fast Learners succeeded in riding the reversed bicycle by DAY 9, their performance on the normal bicycle at the end of practice was worse than before practice: comparison of power spectra for handlebar oscillations between normal bicycle test trials on DAY 1 and on DAY 9 show an increase in oscillation power for the whole frequency range from 0.5 to 4 Hz (*p* < 0.05; paired-sample *t*-test with corrections for multiple comparisons via permutations; see Fig. 4E and Supplementary Fig. 2C for individual oscillation data and example in Fig. 4D).

To compare the performance between the Experienced riders on the normal bicycle (after 10 min of practice) to that of the naïve Novices before they practiced with the reversed bicycle, we compared the power spectra of the handlebar oscillations between these participants. All Experienced riders showed significantly greater oscillation power than all Novices at 0.5 Hz (Fig. 4F); at 1 Hz, two Experienced riders showed greater power than all 10 Novices, a third Experienced rider showed greater power than 9 Novices, and the last showed greater power than 5 Novices. In addition, such increases in oscillations at low frequencies are often well visible upon switching to the normal bicycle: after 5 trials on the reversed bicycle on DAY 3, Experienced riders (notably riders 1, 2, and 3) show an increase in oscillations at the onset of the first trial (see Supplementary Fig. 4). Note that, similarly, upon switching back to the reversed bicycle, two riders show oscillations at the onset of the first trial—results not shown). In summary, the Experienced riders’ performance on the normal bicycle was worse than that of the novice bicycle riders at baseline, at least in part because they exhibited large oscillations soon after switching bicycles.

## Discussion

In laboratory settings, motor learning is often studied using arm-reaching tasks in which subjects must adapt to novel visual or physical perturbations by remapping sensorimotor expectations or contingencies. These paradigms often do not account for the complexity of learning new motor skills in everyday human activities, e.g.^14–18,20–26,30,31^. Here, we studied the short- and long-term acquisition and expression of a complex skill, riding a reversed bicycle, in individuals who had previously learned how to ride normal bicycles. We first discuss our results and then discuss how recent computational motor learning studies can shed light on these findings.

### Learning to ride the reversed bicycle is slow and variable, implicit, and follows an S-shape pattern

Experiment 1 showed that, for participants who already know how to ride a normal bicycle, learning how to ride the reversed bicycle is possible, although challenging and highly variable among learners (see^20^ for similar high variability in learning a different complex motor task). Whereas three-fourths of the Novice participants significantly improved performance after 80 min of practice, only half succeeded in riding the reversed bicycle for at least 20 m. However, with additional practice, riders become experienced. Experiment 2 showed that all four participants who practiced extensively with the reversed bicycle exhibited smaller handlebar oscillations than the Fast Learners at the end of practice. Experienced riders could ride both bicycles for long distances without falling and exhibited similar performance (i.e., handlebar oscillation power) with both bicycles.

Unlike motor adaptation and “de-novo” learning in typical laboratory learning experiments (e.g.,^31–35^), which follows concave (negatively accelerated) learning curves, learning the reversed bicycle initially follows an S-shaped curve, with an inflection point after which learning accelerates (except for the slow learners, for whom performance never significantly improved in the eight sessions). Whereas S-shape learning has been proposed as the hallmark of skill learning, it has only seldom been observed^25,26^. In addition, in some examples of S-shape learning, learning accelerated after the participants discovered the rule, e.g.,^26^. In contrast, despite being instructed and shown how to ride the reversed bicycle, our participants could not ride the reversed bicycle in the initial practice session. This result supports the view that the acquisition of reversed bicycle mapping, at least for participants who already know how to ride a normal bike, is due to implicit learning with no explicit knowledge, in line with previous work suggesting that procedural learning can proceed independently of strategy^34,36^. The lack of any correlation between learning and the three measures of cognition is consistent with this view. Future experiments with participants who do not know how to ride the (normal) bicycle to start with are needed to test the generalizability of these results.

### Experienced riders can switch between bicycles without any failed attempts but exhibited long-term interference effects

Experienced riders could ride both bicycles without any failed attempts in any of the 20-m tests, and then, as we often observed (but not measured), often much farther. However, when measuring the handlebar rotation on the normal bicycle, Experienced riders could not ride the normal bicycle as skillfully as Novice riders before practicing riding the reversed bicycle, including those who reported not having ridden a bicycle in the past year. Thus, Experienced riders could not immediately switch back to skillful performance on a normal bicycle, demonstrating that even a large amount of practice with the reversed bicycle was insufficient to suppress all interferences completely.

### Learning to ride the reversed bicycle requires “unlearning” how to ride the normal bicycle

The Novice participants of Experiment 1 exhibited strong task interference on DAY 5 and DAY 9, as shown by multiple failed attempts to ride the normal bicycle. This result aligns with previous task interference studies in which participants learned a first motor task and then a second reversed version of the task^19,37,38^, see also^18^. Initial practice with the reversed bicycle first increased the degree of interference, as shown by the ascending portion of the inverted U curve in Fig. 4A,B. Then, further practice reduced the interference effect, as shown by the descending part of the inverted U curve in Fig. 4A,B. However, a striking finding of our study is that the Slow Learners, besides their inability to ride the reversed bicycle after 8 days of practice, could not ride the normal bicycle either, often to their great frustration.

We note, however, that none of the participants needed to re-learn how to ride the normal bicycle from scratch while training with the reversed bicycle, as riding the normal bicycle becomes possible again after a “handful of trials”—see also^39^. This suggests that they have not forgotten the skill of normal bicycling riding but that the expression of the skill is deficient.

### Latent and proper skill learning provide a mechanistic account of our results

Recent computational models propose that learning multiple motor skills is due to creating, selecting, and updating control “modules”^32,40,41^. Following memory creation, the expression of the skills for the appropriate task is supported by selecting the corresponding motor modules. Both module selection and update depend on “responsibility signals”. Before the movement onset, the responsibility signals are driven by contextual signals, which rely on sensory cues and the history of the experience with a particular task. During (and following) movements, responsibility signals are updated by task feedback^40^. In other words, before the action, specific modules may be given priority based on their associations with a given sensorimotor context and history, but after the action has been initiated, initial predictions are tested against incoming feedback and then updated. The inference of the appropriate context for each task has been recently called “latent learning”, in contrast to “proper learning”, which is learning the skill itself^32^.

The difficulty and long duration of learning to ride the reversed bicycle suggest that learners create and update a new module and do not simply reuse and adapt the previous bicycle control modules. Unlike in motor adaptation tasks, in mirror tasks in which left and right movements are reversed (as in the reversed bicycle), the error signal between the intended and actual movement direction cannot simply be used to update the motor command from an existing control module, suggesting that a new module needs to be created. Indeed, such “de novo” learning in mirror tasks has been demonstrated to be slower than motor adaptation and likely due to the creation of a new motor module^30,31^.

If initial sensory context and the history of experience with particular tasks are essential for determining which motor modules to select for the upcoming action, then appropriate selection of the new module is initially difficult for the reversed bicycle learners for two reasons: the long history of riding the normal bicycle and, except for the gear reversal for the reversed bicycle, the similarity between the two bicycles used in our experiments (in weight, height, design, color, etc.). In fact, until the first turn, which potentially creates a large sensory prediction error if the “wrong” module is selected, the sensory inputs of both bicycles largely overlap. Thus, early in practice, the overlapping “bicycle” sensory cues and the previous history of normal bicycle riding strongly favor the expression of the motor module for the normal bicycle via a large responsibility signal, making it impossible to ride the reversed bicycle.

With additional practice, our data with slow learners show that a period of “unlearning” of normal bicycle riding precedes the initial learning of riding the reversed bicycle. This suggests that the responsibility signals activate both (incompatible) modules. As a result, proper learning cannot begin, and neither bicycle can be ridden. However, once the responsibility for the reversed bicycle becomes relatively strong (and for the normal bicycle relatively low), the “discovery phase” ends, and proper learning to control this bicycle can proceed. Thus, to learn the reversed bicycle, latent contextual learning occurs first before any proper skill learning is possible.

Because it requires an update and de-update of the same motor modules, motor adaptation (at least when no explicit cues are associated with the perturbation and for small perturbations, see^41^) induces long-lasting aftereffects. In contrast, the aftereffects shown while attempting to ride the normal bicycle while learning how to ride the reversed bicycle last only a few trials and were more pronounced in the initial acquisition phase. Similar short aftereffects in similar reversal tasks have been observed previously^39^, although not in all participants in a mirror tracking task^30^. Thus, because riding the normal bicycle while learning how to ride the reversed bicycle becomes possible after a few trials, latent learning of the appropriate context appears to be faster than proper skill learning. Taken together, our results align with previous work showing that learning to predict is faster than learning to control^42^.

Extensive practice allows separate contexts for both bicycles to be inferred from the sensory feedback obtained as each bicycle is ridden, yielding separate responsibility signals that can select and update the proper motor module for reversed bicycle learning. However, our data show that the Experienced riders’ performance on both bicycles is still degraded despite substantial amounts of practice. This may be because latent learning is not complete even with extensive practice. Because the sensory cues associated with both bicycles are overlapping, and therefore selection based on visual contextual difficult even for Experienced riders, the single large oscillation trials shown by most Experienced riders just after switching to the other bicycle (Supplementary Fig. 5) may be the feedback cue that allows switching between the two motor programs. In a previous study of motor adaptation^41^, we showed that, following learning, a single “trigger trial” can select the appropriate controller via a sudden decrease of the sensory prediction error—see also^32^. Although this requires verification in an additional study, these single large oscillation trials appear akin to trigger trials in selecting the appropriate controller for the current bicycle. It is possible that greater differences between the two bicycles (such as different weights or heights) would be more effective in reducing these long-lasting interferences.

In summary, our data, in light of previous literature, suggest three phases in learning the reversed bicycle. Initially, as the responsibility signals activate the normal bicycle module, riding the reversed bicycle is essentially impossible, despite explicit instructions. Then, as the responsibility signals activate both modules, riding either bicycle is impossible, resulting in an apparent unlearning phase of the normal bicycle. Finally, as the responsibility signals become more specialized to each bicycle, riding both is possible as the module for the reversed bicycle is updated and selected, and the module for the normal bicycle can be selected again. However, because of the large overlap in sensory cues as the riders start on either bicycle, initial sensory feedback, in the form of a few handlebar oscillations, helps select the appropriate module.

### Limitations and future work

As our study is the first to characterize the process of learning and unlearning how to ride the reversed bicycle, it suffers from limitations that can be addressed in future work. First, our Experiment 2 was, at least in part, a natural and not preplanned experiment, in which we used a small convenient sample of experienced riders. We use this approach because learning a new complex skill proficiently takes hours of practice, making the study of the development of expertise difficult to study in the lab. Note that three of these riders (the co-authors) were practicing riding the reversed bicycle as a hobby, before the study was designed, limiting possible bias during learning. However, because they continued to practice during the study, we cannot exclude the possibility that knowledge of procedures and objectives could have resulted in them undertaking some practice strategies that would not be representative of a general population. It is worth noting, however, that explicit instructions regarding the task at hand had little or no benefit, thus knowledge related to abstract study goals seems unlikely to impact skill acquisition. Nonetheless, future work should study a larger sample of participants, naïve to the goals of the study. Second, we instrumented the bicycles with a single sensor, a gyroscope, to measure handlebar rotation velocity. Future studies with fully instrumented bicycles (e.g., tilt sensor on the frame, linear accelerometer on the frame, pedal force sensor, accurate odometer, helmet-mounted gyroscope) are needed to understand whether learning is due to improved feedforward control, feedback control, or both^35,43^. Third, we only recorded gyroscope data during successful test trials. We did not measure speed during practice: thus, we cannot conclude from our data whether participants who went further also went faster, which is related to stability, as faster riders would need smaller wheel turns to maintain stability. Fourth, because we did not measure performance during the 10 min of practice periods (e.g., how many times participants put their foot down to stabilize, the actual time of riding, or the total distance covered), the precise relationship between the parameters of practice and daily improvements is unknown. For instance, the Fast Learners probably covered much longer distances during practice. As a result, the effective “dosage” of practice may have been greater for those learners. Finally, future virtual reversed bicycle experiments may be designed to dissociate learning how to steer from learning how to maintain balance.

## Methods

### Experimental design

In Experiment 1, we instructed 20 novice participants to practice riding the reversed-steering bicycle in eight practice sessions. Participants completed the experiment over nine consecutive weekdays: DAY 1–DAY 9 (Fig. 1B). DAY 1 involved inclusion screening, cognitive testing, and normal bicycle testing for five trials of a 20-m straight distance. Then, participants completed eight practice sessions with the reversed bicycle from DAY 1 to 8. Each session involved three phases with the reversed bicycle: (1) a *pre*-practice test of five trials, (2) 10 min of free practice, and (3) a *post*-practice test of five trials. In addition, before the practice on DAY 5 and then on DAY 9, we tested whether learning to ride the reversed bicycle interfered with riding the normal bicycle.

Because these participants were only able to learn to ride the reversed bicycle after initially unlearning how to ride a standard bicycle, we carried out Experiment 2, in which four participants engaged in extended practice over several weeks to assess the ability to ride both types of bicycles. The four Experienced riders performed a series of tests with the reversed and normal bicycle over 3 days (Fig. 1B). On DAY 1 and DAY 2, we assessed whether additional practice improved performance on the normal and reversed bicycle, respectively. On each of these days, the participants performed a five-trial *pre*-practice test followed by 10 min of free practice, and finally a five-trial *post*-practice test. On DAY 3, we assessed their capacity to switch between bicycles in a design identical to DAY 9 in Experiment 1: they performed 5 trials of reversed bicycle, then 5 trials of normal bicycle, and, finally, 5 trials on the reversed bicycle.

### Participants

#### Experiment 1

Twenty healthy volunteers (university graduate students, mean age 26.1 ± 3.1 years, height 171.3 ± 7.8 cm, weight 72 ± 15.4 kg, weekly physical activity 3.4 ± 2 h, 10 females) participated in Experiment 1. All participants reported knowing how to ride a bicycle but had not practiced in the past year. In anticipation of potential interference, people who commute with bicycles or motorcycles were excluded for their safety. Participants were instructed not to ride any other bicycles during the experiment. These participants were naïve about the objectives and design of the study before completing the experiment.

#### Experiment 2

Four reversed bicycle Experienced riders (including co-authors ETS, TM, and JM) (ages between 45 and 25, 3 males) participated in Experiment 2. These participants practiced the reversed bicycle (and normal bicycle) for a minimum of 12 total hours over at least 8 weeks and reported riding proficiently on both the normal and the reversed bicycles, including in busy areas and making sharp turns. Note that all these participants knew about the objectives and design of the study before completing the experiment.

All participants wore helmets during all practice and testing sessions. The University of Southern California Institutional Review Board approved the study, all research was performed in accordance with relevant guidelines and regulations, and all participants provided written informed consent.

### Characteristics of the reversed bicycle

Figure 1A illustrates the handlebar-to-wheel coupling of the normal and the reversed bicycles. Two reversed bicycles allowing adjustable seat height were custom-built for this study—a large bicycle (frame height of 48 cm and a wheel diameter of 66 cm) and a medium bicycle (frame height of 38 cm and a wheel diameter of 60 cm). Two matching normal bicycles were also used. Participants chose one of the two bicycle sizes for the duration of the experiment based on their preferences. An LG Journey™ smartphone was placed horizontally on the handlebar and the phone’s gyroscope data were accessed using the “Physics Toolbox Sensor Suite” application (sampling frequency ~ 500 Hz).

### *Procedures of Experiment 1

#### Reversed bicycle practice

The objective given to the participants was to ride 20 m in a straight line with the reversed bicycle. During the eight 10-min practice sessions, participants received no feedback on their ongoing performance or strategy. However, before the first practice session, we explained to the participants the reversed bicycle characteristics. We showed how to turn the handlebar to control direction and balance according to the reversed tilt to wheel coupling. Participants were given the following instructions “On the normal bike, you need to coordinate the tire’s turn with the bike’s tilt to remain upright, and you control the tire’s turn with the handlebar. On the reversed bike, that same coordination is needed between the tire and the bike, but now you need to turn the handlebars in the opposite direction”.

#### Reversed bicycle performance testing

During pre- and post-practice tests, the participants performed 5 trials with the goal of riding along a straight line for as long as possible, up to 20 m, without touching the ground with either foot. Participants rode the bicycles in the center of an outdoor straight concrete path, of 4-m width, and were not allowed to ride outside of the path, which was bordered by grass. A visible straight line was drawn directly in the center of the concrete path, as well as lines on the edges of the path. The start and finish lines (separated by 20 m) were clearly marked with tape. A trial started when the participants placed both feet on the pedals. The trial ended when one of their feet touched the ground. The experimenter then measured the distance between the starting line and the end position of the bike using a standard surveyor's wheel. The gyroscope recorded the handlebar rotation velocity (i.e., yaw). Then, the participant returned to the start line to perform the next trial.

#### Task interference assessment with the normal bicycle

On DAY 5 and DAY 9, after the usual five-trial pre-test with the reversed bicycle, participants performed an interference test with the normal bicycle, with the goal of riding 20 m along a straight line. The number of trials necessary to achieve this goal was recorded to quantify the interference caused by learning the reversed bicycle. Note that there was no upper limit on trials/attempts, and a minimum of five trials was recorded, even in case of success before the fifth trial. Then, participants performed five post-interference assessment trials on the reversed bicycle. On DAY 5, the participants completed the 10-min practice phase and the usual five-trial post-practice test (Fig. 1B). We recorded the distance covered and the handlebar rotation velocity after each trial. Note that there were no structured breaks in between tests of the reversed and normal bikes. Participants were allowed to rest briefly as needed before proceeding with the next trials.

### Statistical analysis

#### Distance covered with the reversed bicycle

Our primary outcome measure was the mean distance (in meters) during each pre- and post-practice test performed with the reversed bicycle from pre-test DAY 1 to pre-test DAY 9.

#### Individual assessment of learning via mixed effect sigmoidal models

We assessed individual learning performance for all individuals simultaneously by fitting the mean distance covered in five trials with the reversed bicycle, in all pre- and post-practice tests of all 8 practice days, and in the pre-test of DAY 9, with a mixed-effect non-linear model. We assumed that the rate of improvement is the same for both pre- and post-practice tests. For each participant *i*, session *j*, the distance is modeled as:

1 $${D}_{i,j}= \mathit{min} \left\{{D}_{max} , \right.\frac{2*{D}_{max}}{(1+ \mathit{exp}\left(-{a}_{i}({t}_{j}- {b}_{i}-{c}_{i} T\right)} + { \epsilon }_{i,j}\}$$

where $${D}_{max}$$ = 20 m; $${t}_{j}$$ is test at session *j*;* T* is a binary variable coding for tests. The model characterizes practice via three mixed-effect parameters for each participant i: (1) a “day to the 20-m criterion” parameters $${b}_{i}$$(the sigmoid threshold), which is the (modeled) number of days until reached 20-m criterion in post-test, (2) a “learning rate” parameters $${a}_{i}$$ (the sigmoid slope), which is the rate of learning at the 20-m criterion$$,$$ and (3) an “overnight forgetting” parameter $${c}_{i}$$ (offset from the threshold), which is the (modeled) difference in number of days to criterion between pre- and post-tests. Note that in this model, we assume that each session is given on 9 consecutive days (whereas in the actual experiment, practice was only given on weekdays). Also note that, even if some participants do not actually reach the 20-m criterion, the model fitting procedure can still estimate the parameter $${b}_{i}$$ based on the data available. We used an unstructured random effect covariance matrix and verified that the unstructured matrix yielded a smaller Bayesian Information Criterion (BIC) compared to a diagonal structure. Fitting was performed with the Matlab function *nlmefit*. We chose to model distance as the non-linearity *min()* and the bottom half of a sigmoid of amplitude $$2*{{\text{D}}}_{{\text{max}}}$$ and not directly as sigmoid of amplitude $${{\text{D}}}_{{\text{max}}}$$ because we noted that participants made slow progress initially, followed by quick progress. In other words, once the participants started to be able to ride the reversed bicycle, they could often ride distances much longer than 20 m. A sigmoid with amplitude $${D}_{max}$$ would predict a decreasing rate of improvement near $${D}_{max}$$. In contrast, the sigmoid of amplitude $$2*{D}_{max}$$ with a minimum non-linearity has a sharp transition with the maximal rate of improvement just before reaching $${D}_{max}$$ (with slope $${a}_{i})$$. We compared this model with an exponential mixed-effect model with initial faster increase and then a slow reduction in performance, as often used in motor learning studies.

#### Determining the end of exploration and identification of sub-groups of learners

To determine if a relationship existed between the discovery of a new control strategy and the beginning of rapid improvement across days, we calculated (1) the change in the average trial distance before vs. after practice each day and (2) the change in the average trial distance between the pre-practice trials each consecutive pair of days (DAY 1 vs 2, DAY 2 vs. 3, etc.). Because no relevant improvement occurred on DAY 1 (pre- to post-practice) or between DAY 1 and 2 (pre- practice trials), we recast each participant’s performance changes over subsequent days as z-scores with respect to the cross-participant mean and standard deviation of either within-day improvement on day 1, or across-day improvement from day 1 to day 2. For each participant, we recorded when the first within-day and across-day z-scores exceeded 1.65 (the 95% confidence level for a 1-sided test). We then performed a linear regression to determine if the first significant within-day improvement predicted the beginning of learning (the first significant across-day improvement).

#### Angular handlebar rotation velocity data

For all reversed and normal bicycle test trials of Experiment 1 in which the distance covered was 20 m, and for all trials of Experiment 2, we analyzed the angular velocity data (in radians per second) associated with the rotation of the handlebar (i.e., yaw, *z*-axis). Angular velocity was recorded at approximately 500 Hz and then interpolated to exactly 500 Hz. Angular velocity was then filtered with a Butterworth filter at 6 Hz. The starting time of each trial was identified by computing the standard deviation of handlebar velocity data in a moving window indexed by *tstart*. When the standard deviation was 10 times greater in the window defined by t > *tstart* than in the window defined by t ≤ *tstart* (baseline), the trial was marked as “start”. A similar procedure was used to identify the end of the trial.

We then performed a frequency analysis of the angular velocity data during the trial duration. We used the *pwelch* function in Matlab, with Hamming window of 1000 samples, and 50% overlap between contiguous sections. Power was computed in dB by taking the 10 × log_10_ of the power spectral density. Difference in power at multiple frequencies (from 0.5 to 4 Hz, in increments of 0.1 Hz) between DAY 5 and DAY 9 was performed using a two-tailed *t*-test in which we controlled for multiple comparisons using the "tmax" permutation method for adjusting the p values in a way that controls the family-wise error rate^44^.

#### Interference assessment with the normal bicycle

During DAY 5 and DAY 9, the number of trials necessary to achieve the 20-m criterion with the normal bicycle was recorded to quantify how learning to ride the reversed bicycle interfered with the ability of the participant to ride the normal bicycle. This interference effect was investigated by assessing the relationship between the cumulative distance in test trials until DAY 5 and DAY 9 and the number of attempts to ride 20 m with the normal bicycle on DAY 5 and DAY 9 (on which we applied a log_10_ transformation because some participants required dozen of attempts). This relationship was modeled with a second-order model, as we predicted that both Slow and Fast learners would require fewer attempts to ride the normal bicycle successfully. Number of trials on DAY 5 and DAY 9 were used in a mixed-effect second-order model with a binary variable coding for days (DAY 5 = 0, DAY 9 = 1), with a random intercept. In a post-hoc analysis, a comparison between second- and first- order models was performed with the likelihood ratio test.

#### Comparison of angular handlebar rotation velocity data between Novice (Experiment 1) and Experienced riders (Experiment 2)

We then performed additional power spectra analyses to compare (1) the performance of the Experienced riders on both types of bicycles, (2) the performance of the Experienced riders to that of the Fast Learner participants at the end of practice on the reversed bicycle, and (3) the performance of the Experienced and Novice participants before practice on the normal bicycle. Methods were as that described above, except that, because of the small number of Experienced riders, we tested for difference in power using one-sample t-tests (e.g., at each frequency from 0.5 to 4 Hz, the power of each Experienced rider was compared to that of the Novice group). As above, we controlled for multiple comparisons using the "tmax" permutation method for adjusting the p values in a way that controls the family-wise error rate.

### Supplementary Information

Supplementary Information.

## Data Availability

All data, code, and materials used in the analyses are available for purposes of reproducing or extending the analyses by request to the corresponding author.
