# Supplementary material for: Initial development of skill with a reversed bicycle and a case series of experienced riders
Source: Sci Rep. 2024 Feb 21;14:4334. doi: 10.1038/s41598-024-54595-8 (PMC10881966; doi:10.1038/s41598-024-54595-8)

**Supplementary materials**

**Supplementary Table 1**. Experiment 1: Individual initial and final reversed bicycle distances, interference on the normal bicycle, and day to reversed bicycle 20-meter criterion (when applicable).

|  | **Reversed bicycle** | | | | **Normal bicycle** | |
| --- | --- | --- | --- | --- | --- | --- |
| Participant | Initial distance  DAY 1 *pre*-test  (*m*) | Day 20-meter  Criterion Reached | Total 20-meter trials  (*/ 50 trials*) | Final distance  DAY 9 *pre*-test  (*m*) | Interference  (*number of trials*) | |
|  |  |  |  |  | DAY 5 | DAY 9 |
| **9** | 1.9 ± 0.3 | DAY 4 | 33 | 20 ± 0 | 2 | 2 |
| **7** | 1.7 ± 0.5 | DAY 5 | 27 | 20 ± 0 | 8 | 4 |
| **5** | 2 ± 0.4 | DAY 5 | 22 | 20 ± 0 | 4 | 3 |
| **2** | 1.6 ± 0.4 | DAY 5 | 24 | 20 ± 0 | 12 | 13 |
| **1** | 1.3 ± 0.3 | DAY 5 | 11 | 14.9 ± 7.7 | *_NA_* | 35 |
| **13** | 1.3 ± 0.4 | DAY 5 | 15 | 19.7 ± 0.8 | 1 | 2 |
| **20** | 1.7 ± 0.6 | DAY 7 | 10 | 20 ± 0 | 4 | 3 |
| **14** | 1.9 ± 0.3 | DAY 7 | 11 | 20 ± 0 | 4 | 4 |
| **10** | 1.1 ± 0.3 | DAY 8 | 6 | 18.8 ± 2.1 | 4 | 14 |
| **6** | 1.6 ± 0.4 | DAY 6 | 11 | 20 ± 0 | 4 | 13 |
| **17** | 1.4 ± 0.3 | DAY 9 | 4 | 19.8 ± 0.5 | 29 | 19 |
| **4** | 1.3 ± 0.3 | DAY 9 | 3 | 18.3 ± 2.9 | 1 | 33 |
| **3** | 1.2 ± 0.4 | DAY 9 | 2 | 12.2 ± 7.5 | 3 | 6 |
| **16** | 1.2 ± 0.1 | **🗶** | **🗶** | 13.1 ± 4.6 | 1 | 12 |
| **18** | 1.9 ± 0.5 | **🗶** | **🗶** | 7.1 ± 1.1 | 1 | 3 |
| **8** | 1.1 ± 0.1 | **🗶** | **🗶** | 6.9 ± 1.5 | 2 | 15 |
| **11** | 1.4 ± 0.6 | **🗶** | **🗶** | 5.1 ± 2.7 | 1 | 12 |
| **15** | 1 ± 0.2 | **🗶** | **🗶** | 2.3 ± 0.6 | 2 | 10 |
| **12** | 1.2 ± 0.2 | **🗶** | **🗶** | 3.3 ± 0.4 | 1 | 4 |
| **19** | 1.4 ± 0.4 | **🗶** | **🗶** | 2.9 ± 0.9 | 1 | 1 |

*Notes*. Initial and final distances are expressed as the mean ± SD of 5 trials. For the interference, the number of trials required to ride with the normal bicycle throughout the 20-meter straight line criterion without touching the ground with either foot is reported. *NA*: non-applicable. Participants’ data are arranged in increasing order of their Learning Day parameter according to the following sub-groups: 10 Fast-learners participants (in **green**): 5 Moderate-learners participants (in **orange**): and 5 Slow-learners participants (in **red**):; **🗶**: participant did not achieve the 20-meter criterion.

**Supplementary results: Learning the reversed bicycle mapping is not predicted by cognitive measures.**

We investigated whether the shape of reversed bicycle learning is predicted by the cognitive performances of the participants of Experiment 1. On DAY 1, all participants (except for S3) were given three cognitive tests: the Stroop test to measure cognitive interference, that is, the capacity to maintain a course of action in the face of intrusion by other stimuli^35^, the Trail Making Test to measure mental flexibility^36^, and the forward and backward Digit Span Task to measure working memory^37^.

In line with our hypothesis that learning the reversed bicycle is an implicit learning process, we tested for the null hypothesis of a correlation between the cognitive performances tested and the learning parameters aforementioned: (1) “day to 20-meter criterion” parameters $b_{i}$, (2) “learning rate” parameters $a_{i},$and (3) “overnight forgetting” parameters $c_{i}$.of the sigmoidal mixed effect model (see equation 1 in Methods). Indeed, there was no correlation between cognitive tests and the learning rate $a_{i}$ , the learning to criterion $b_{i}$ or the learning rate parameters $c_{i}$ of the model (Person correlations, all *p* > 0.4).

**Supplementary Figure 1.** Relationship between the first day of within-day performance and the first between-day performance change (“learning”) for the 15 participants of experiment 1 who showed at least one significant within-day performance change. These participants were classified as Fast or Moderate learners. See text.


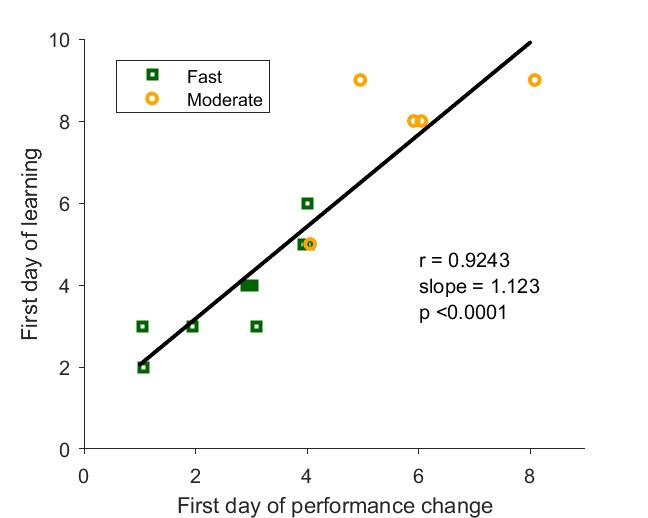


**Supplementary Figure 2.** Handlebar oscillations for the 10 participants in the Fast Learners sub-group in Experiment: From left to right: Handlebar rotation velocity for the first (dark blue) and the last (light blue) 20-meters reversed bicycle trial, and Handlebar rotation velocity for the first (red) and the last (purple) 20-meter normal bicycle trial. x-axis: time in seconds, and y-axis: oscillations amplitudes in radians/seconds.

**
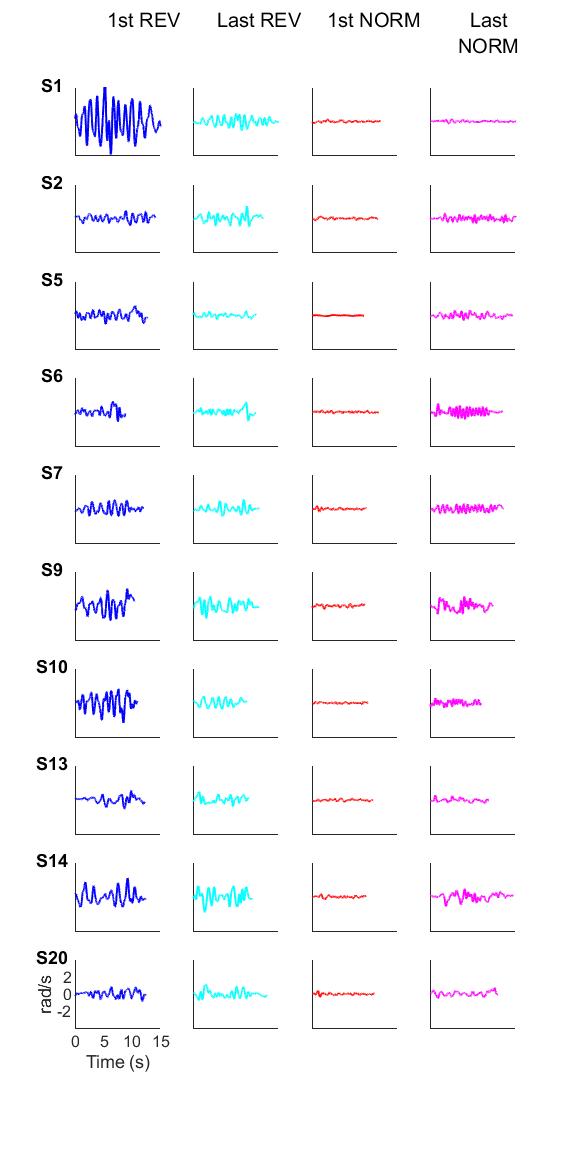
**

**Supplementary Figure 3.** Comparison of handlebar oscillations on both reversed and normal bicycles for the four Experienced riders in Experiment 2. A. Comparison of the oscillations on the reversed bicycle on the last trial of Day 1 and on the normal bicycle on the last trial of Day 2. B. Power spectra for both bicycles of all four Experienced riders; there were no significant differences in power spectra for any frequencies from 0.5 to 4 Hz (all p > 0.05).


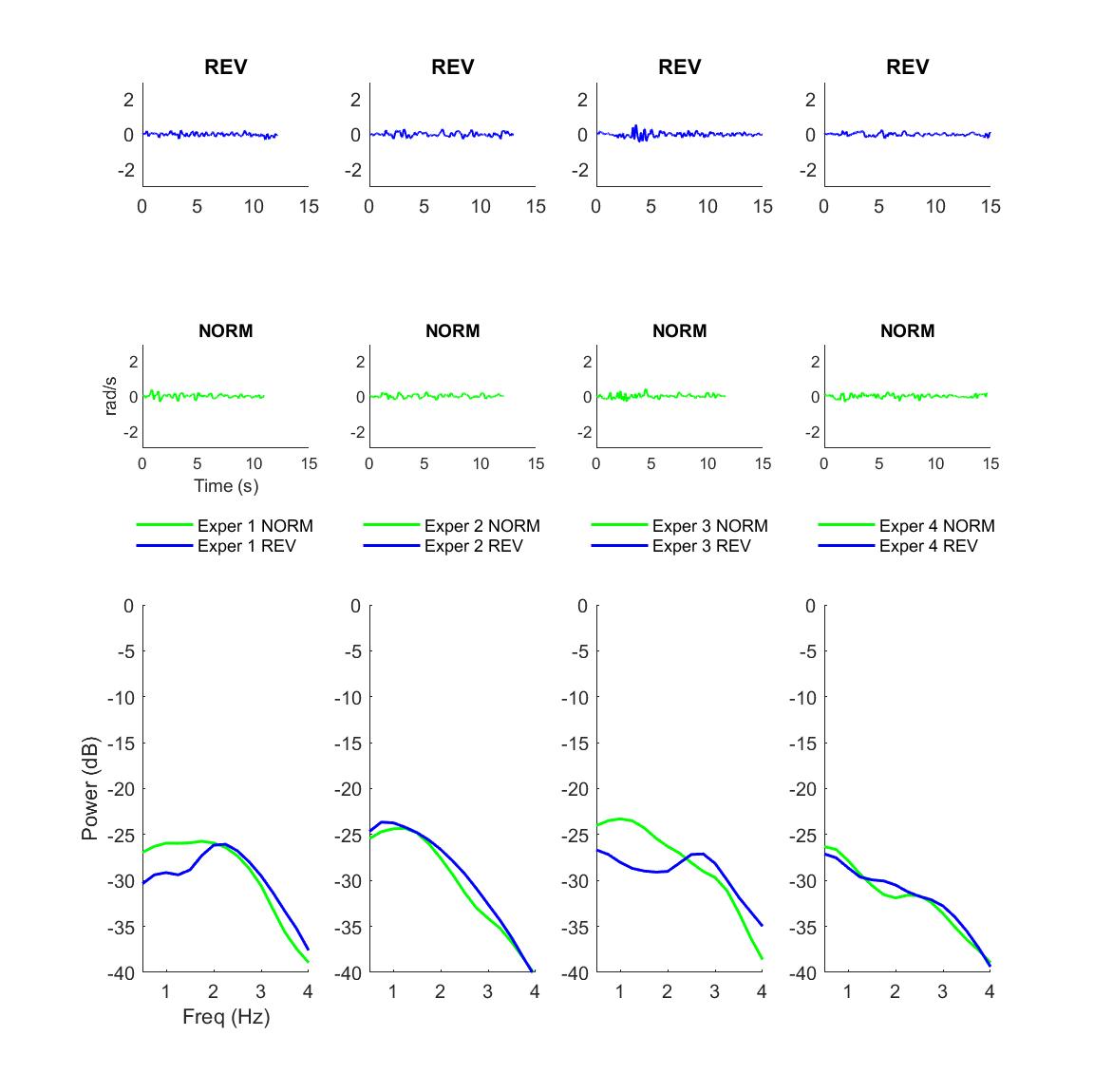


**Supplementary Figure 4.** Handlebar oscillations for the 5 trials on the normal bicycle performed just after riding 5 trials on the reversed bicycle for all four Experienced riders (Experiment 2, DAY 3). Note the initial large oscillations in Experienced riders 1, 2, and 4.


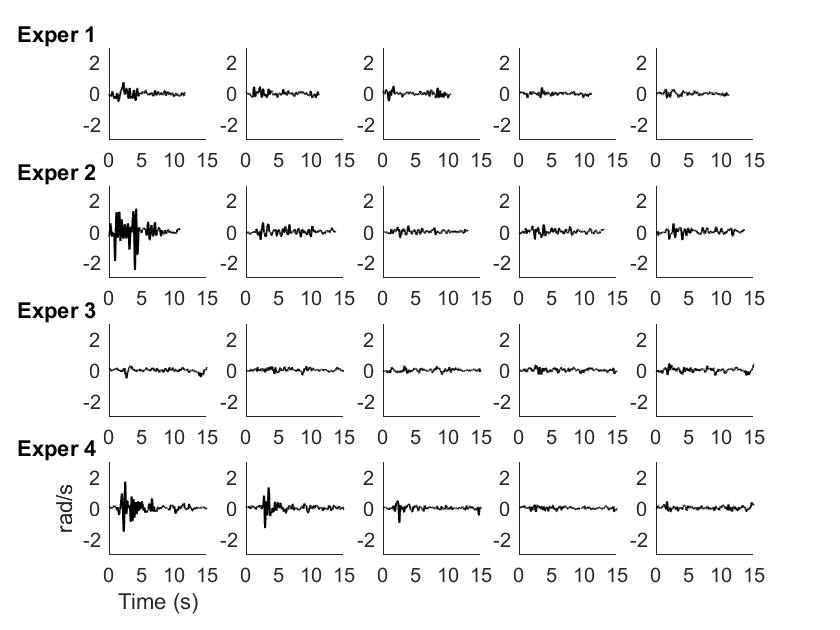

Supplement: Supplementary file 1 — Supplementary Information. [file 41598_2024_54595_MOESM1_ESM.docx]
